# Targeted Lipid Analysis of Haemolytic Mycelial Extracts of *Aspergillus niger*

**DOI:** 10.3390/molecules19079051

**Published:** 2014-06-30

**Authors:** Maruša Novak, Kristina Sepčić, Nada Kraševec, Igor Križaj, Peter Maček, Gregor Anderluh, Graziano Guella, Ines Mancini

**Affiliations:** 1 Department of Biology, Biotechnical Faculty, University of Ljubljana, 1000 Ljubljana, Slovenia; E-Mails: marusa.novak@bf.uni-lj.si (M.N.); kristina.sepcic@bf.uni-lj.si (K.S.); peter.macek@bf.uni-lj.si (P.M.); 2 National Institute of Chemistry, 1000 Ljubljana, Slovenia; E-Mails: nada.krasevec@ki.si (N.K.); gregor.anderluh@ki.si (G.A.); 3 Department of Molecular and Biomedical Sciences, Jožef Stefan Institute, 1000 Ljubljana, Slovenia, E-Mail: igor.krizaj@ijs.si; 4 Department of Chemistry and Biochemistry, Faculty of Chemistry and Chemical Technology, University of Ljubljana, 1000 Ljubljana, Slovenia; 5 Department of Physics, Bioorganic Chemistry Laboratory, University of Trento, Via Sommarive 14, I-38123 Povo-Trento, Italy; E-Mail: graziano.guella@unitn.it; 6 Biophysical Institute, CNR, via alla Cascata 56/C, I-38123 Povo, Trento, Italy

**Keywords:** *Aspergillus niger*, haemolytic activity, fatty acids, lipid composition, liquid chromatography-selectrospray ionisation tandem mass spectrometry

## Abstract

Ethanolic extracts of mycelia from *Aspergillus niger* (strain N402) grown in liquid media were observed to have haemolytic activity on bovine erythrocytes. This haemolytic activity decreased significantly during the time of growth (1–3 days). Moreover, when *A. niger* was grown on carbon-deprived medium, the efficiency of this haemolytic activity in the ethanolic extracts was much lower than when grown in carbon-enriched medium, and became almost undetectable after 3 days of growth in carbon-deprived medium. The lipid composition of these ethanolic extracts was analysed by liquid chromatography–electrospray ionisation tandem mass spectrometry. This haemolytic activity can be mainly linked to the relative levels of the molar ratios of the unsaturated fatty acids and lysophosphatidylcholines.

## 1. Introduction

Filamentous fungi of the genus *Aspergillus* comprise more than 200 very common and widespread species, including *Aspergillus niger* [1]. Members of this genus have important roles in natural ecosystems and in industry, where they are used for the production of numerous enzymes, organic acids, and secondary metabolites. However, some are known as causative agents of diseases in plants and/or animals, and can therefore also have a negative impact on the human economy. 

*Aspergillus niger* is a saprophyte that is commonly isolated from soil, dead leaves, and other decaying plant matter [2]. It reproduces asexually through spores, which can be found almost everywhere. For many decades, *A. niger* has been a very important biotechnological organism, as it has been used for the production of citric and gluconic acids, as well as for many extracellular enzymes, such as pectinase, protease and glucoamylase. As *A. niger* can efficiently produce native proteins, this species is also a common host for the production of heterologous proteins. Industrially important products of *A. niger* are generally recognised as safe by the United States Food and Drug Administration. 

Upon inhalation of the spores, some *Aspergillu*s spp. (mainly *Aspergillu*s *fumigatus*, *Aspergillu*s *flavus*, *A. niger* and *Aspergillu*s *terreus*) can cause human diseases, especially in immuno-compromised patients [3,4,5]. *A. niger* has to date been identified as the causative agent of asthma, allergy, aspergilloma and aspergillosis. Generally, these diseases are related either to prolonged exposure to large quantities of spores, or to immune deficiency. In the tropics, *A. niger* is known to cause infection of the external ear canal, which is known as otomycosis. It is also a known food and feed contaminant, which is found especially on grapes, coffee, peanuts, onions, and maize [6,7]. This can be harmful for human and animal health, because of the production of the carcinogenic mycotoxins ochratoxin A [8] and fumonizin B2 [9]. 

The virulence of *Aspergillus* spp. has been shown to be associated with the production of various toxic compounds, which include cell-wall components, allergens, pigments, adhesins, enzymes and toxins, and these appear to act in synergistic/additive ways [10,11]. However, none of these molecules has yet been established specifically as a virulence factor. Some of these compounds can act as haemolysins, and one such example is Asp-haemolysin, a haemolytic 15-kDa protein that was isolated from *A. fumigatus* [12,13]. Asp-haemolysin belongs to the large protein family of aegerolysins (PF06355; IPR 009413) [14,15]. More than 300 aegerolysin proteins, or their nucleotide sequences, have been described to date in different fungi, bacteria and plants. Aegerolysins appear to have pleiotropic roles in the organisms that produce them, and some fungal representatives have been shown to be associated with haemolysis [16].

During our initial search for haemolytically active aegerolysin-like proteins and other haemolytic compounds in various *Aspergillus* spp., *A. niger* N402 was found as one of the strains that have haemolytic activity on sheep blood agar plates. Two aegerolysin-like proteins were identified in the genome of this fungus. One of these, An01g09980, has 49% identity to the abovementioned Asp-haemolysin from *A. fumigatus*, and it has been detected in considerable amounts in the secretome of *A. niger*, with its expression particularly high after exhaustion of the carbon source in liquid medium [17]. Both aegerolysin-like proteins were also isolated as recombinant proteins, but these showed no haemolytic potential (Maruša Novak, Biotechnical Faculty, University of Ljubljana, personal communication). On the other hand, ethanolic extracts of these *A. niger* mycelia were haemolytic on bovine erythrocytes. To the best of our knowledge, there have not been any reports on haemolytically active organic molecules in *Aspergillus* spp. However, free fatty acids and some other phospholipid derivatives, but not sterols, have been reported to have haemolytic and cytotoxic effects in some other fungal species [18,19]. Thus, in the present study, ethanolic extracts from *A. niger* N402 mycelia grown under different conditions and showing different degrees of haemolytic activities, were analysed for their lipid composition using liquid chromatography–electrospray ionisation tandem mass spectrometry (LC–ESI-MS/MS). We report here how a variation in the carbon source in the fungal growth medium and the relative changes in the production of fatty acids, lysophosphatidylcholines and diacylglycerols are related to this haemolytic activity.

## 2. Results and Discussion

### 2.1. Haemolytic Activity of Mycelial Ethanolic Extracts of Aspergillus niger N402

**Figure 1:**
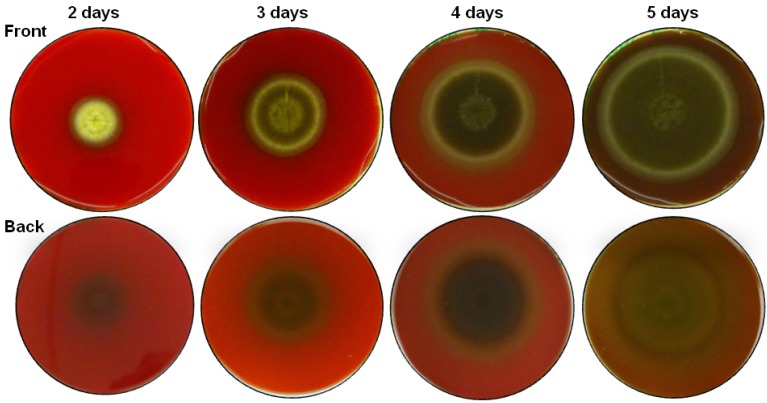
Front and back views of *Aspergillus niger* N402 mycelia grown for 2–5 days on tryptic soy agar supplemented with 5% defibrinated sheep blood. Partial haemolysis (dark and greenish agar) is observed around and underneath the colony.

**Figure 2:**
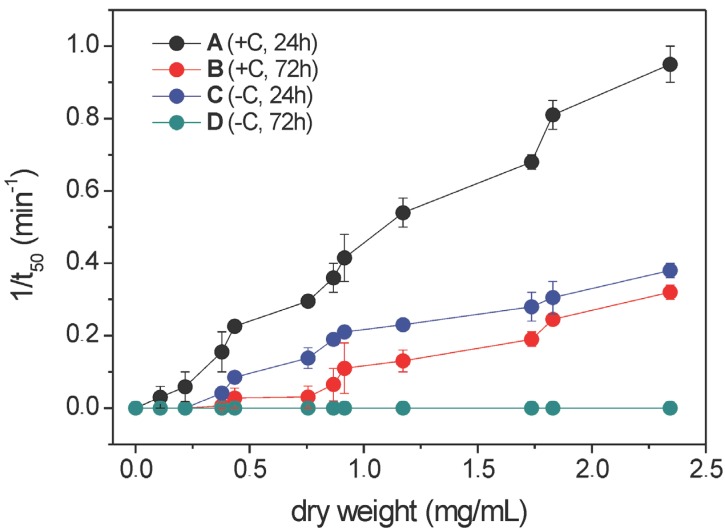
Haemolytic activity of ethanolic extracts from *Aspergillus niger* N402 grown under different conditions (samples A–D, as indicated). t_50_, time to induce 50% lysis of bovine erythrocytes. Data are means ± standard error from three independent biological replicates. +C, growth in the presence of glucose; −C, growth without glucose.

During the growth of *A. nig*er N402 on tryptic soy agar with 5% (v/v) sheep blood, we observed partial haemolysis around the mycelia (dark and greenish agar underneath the colony, Figure 1), which indicated that *A. nig*er N402 was producing haemolytically active compounds. Aqueous extracts of *A. nig*er N402 mycelia grown in liquid minimal medium with glucose or without it were not haemolytic, which suggested that this biological activity was not linked to the presence of water-soluble proteins or other polar compounds. On the contrary, ethanolic mycelial extracts were haemolytically active on bovine erythrocytes. These ethanolic extracts that were evaluated for their haemolytic activity were also obtained from mycelia grown in liquid minimal medium for 24 h or 72 h with (+C) or without (−C) glucose as the carbon source in the medium, which are here denoted as: sample A, N402 +C, 24 h; sample B, N402 +C, 72 h; sample C, N402 −C, 24 h; and sample D, N402 −C, 72 h. The analysis of the haemolytic activities of these ethanolic extracts from *A. nig*er N402 grown under each of these conditions (*i.e.*, ±glucose, 24/72 h) were carried out as three repetitions, with identical trends and similar haemolytic activities obtained. As illustrated in Figure 2, monitoring of the haemolytic activity of the raw ethanolic extracts at 1 mg/mL showed a reciprocal half-time of haemolysis of sample A of 0.45 min^−^^1^, while after 3 days of growth with glucose (sample B), the haemolysis was some four-fold lower. At the same concentration, the haemolytic activity of extract C (growth without glucose) was approximately two-fold lower compared to sample A, while for the longer growth without glucose of sample D, the haemolytic activity had totally disappeared. These data indicated that the haemolytic activity of these ethanolic extracts from *A. nig*er N402 decreases with the time of growth, and with growth in medium without glucose.

### 2.2. Lipid Profile of Ethanolic Extracts from Aspergillus niger N402 Using LC–ESI-MS/MS

**Figure 3:**
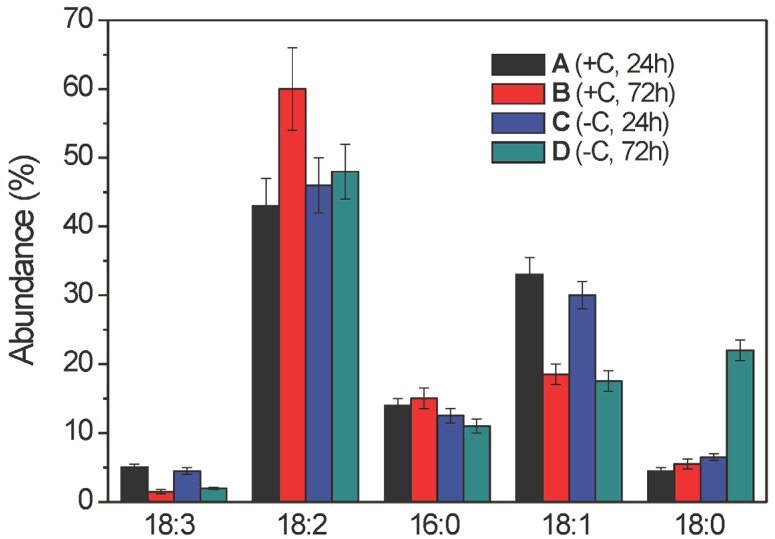
Intra-sample distribution of free fatty acid lipid species (as % abundances) of the ethanolic extracts of samples A–D (as indicated) from the mycelia of *Aspergillus niger* N402, as obtained by LC–ESI(−)-MS analysis. The fatty acid structures along the x-axis are shown according to their increasing retention times (see Table 1) and the % abundance values along y-axis are shown as % molar fractions of each species within a given sample. Data are means ± relative uncertainty error from three independent biological replicates. +C, growth in the presence of glucose; −C, growth without glucose.

**Figure 4:**
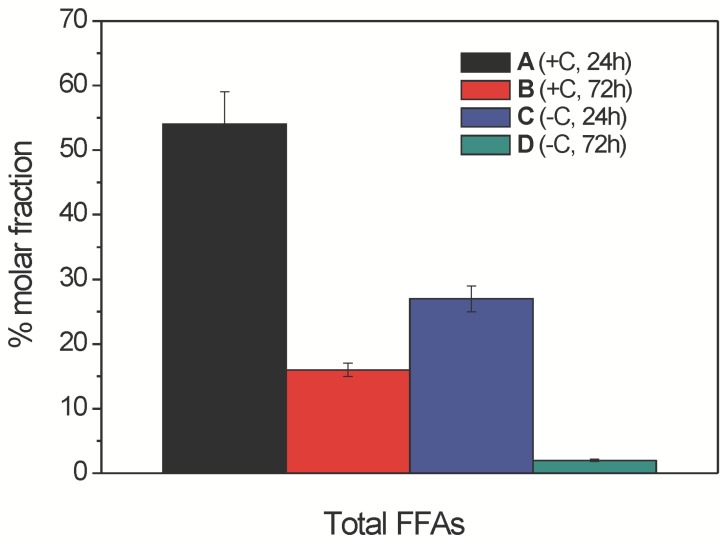
Inter-samples distributions of total free fatty acids (as % molar fractions) in the ethanolic extracts from samples A–D (as indicated) from the mycelia of *Aspergillus niger* N402, as obtained by LC–ESI(−)/MS/MS analysis. Data are means ± relative uncertainty error from three independent biological replicates. +C, growth in the presence of glucose;−C, growth without glucose.

**Figure 5:**
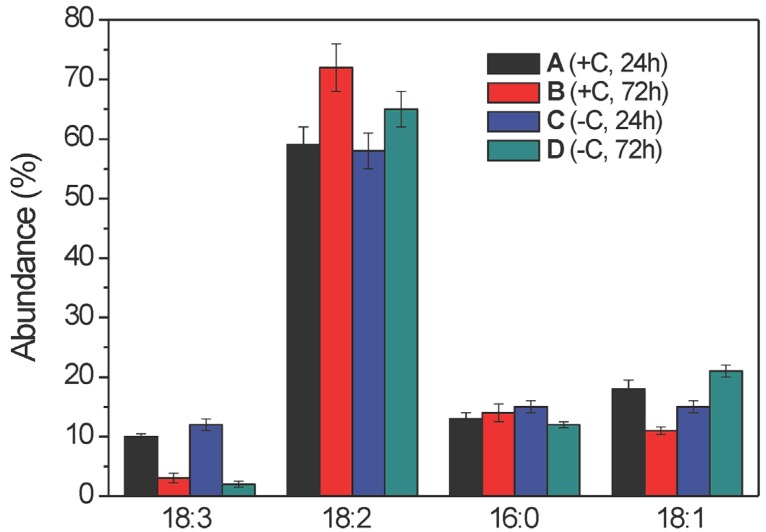
Intra-sample distribution of lysophosphatidylcholine lipid species (as % abundances) in the ethanolic extracts of samples A–D (as indicated) from the mycelia of *Aspergillus niger* N402, obtained by LC–ESI(+)-MS analysis. The fatty acid structures along the x-axis are shown according to their increasing retention times (see Table 2) and the abundance values along y-axis are shown as % molar fractions of every lysoPC species within a given sample. Data are means ± relative uncertainty error from three independent biological replicates. +C, growth in the presence of glucose; −C, growth without glucose.

**Figure 6:**
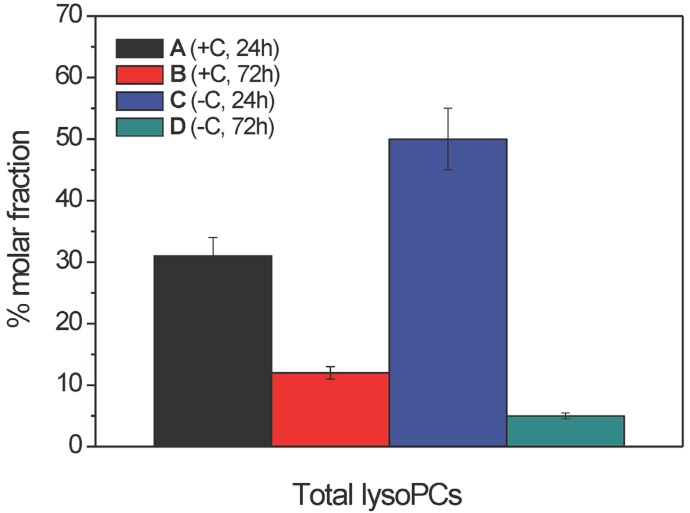
Inter-sample distribution of total lysophosphatidylcholines (as % molar fractions) in the ethanolic extracts of samples A–D (as indicated) from the mycelia of *Aspergillus niger* N402, as obtained by LC–ESI-(+) MS/MS analysis. Data are means ± relative uncertainty error from three independent biological replicates. +C, growth in the presence of glucose; −C, growth without glucose.

**Figure 7:**
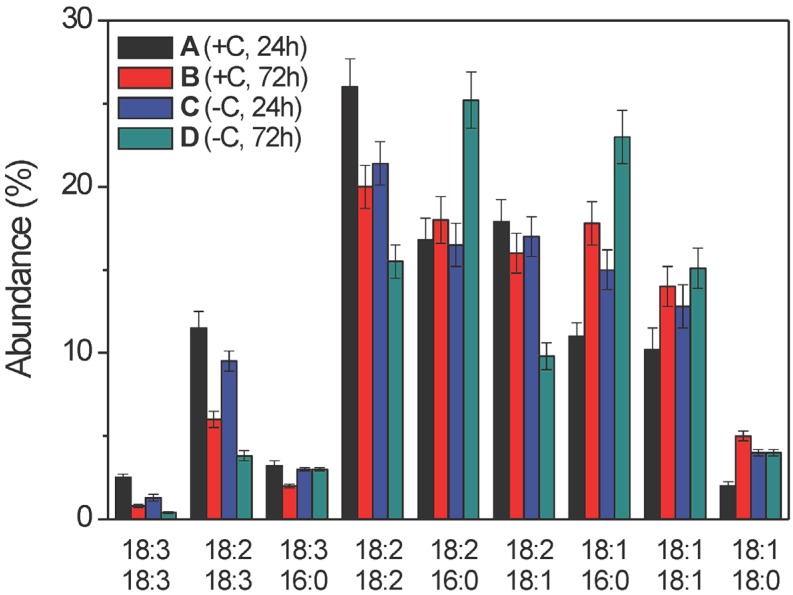
Intra-sample distribution of diacylglycerol acyl chains (as % abundances) in the ethanolic extracts of samples A–D (as indicated) from the mycelia of *Aspergillus niger* N402, obtained by LC–ESI(+)-MS analysis. The fatty acid structures along the x-axis are shown according to their increasing retention times (see Table 3), and their abundance up the y-axis are shown as % molar fractions of every DAG species within a given sample. Data are means ±relative uncertainty error from three independent biological replicates. +C, growth in the presence of glucose; −C, growth without glucose.

**Figure 8:**
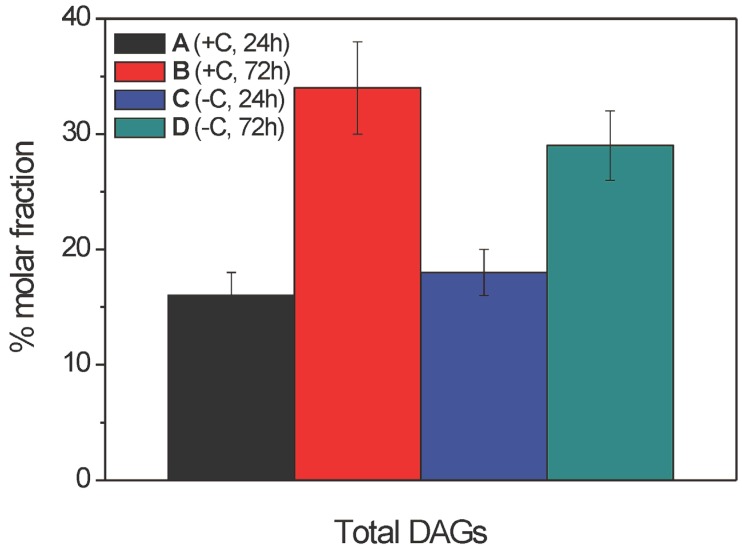
Inter-sample distribution of total diacylglycerols (as % molar fractions) in the ethanolic extracts from samples A–D (as indicated) from the mycelia of *Aspergillus niger* N402, as obtained by LC–ESI-(+) MS analysis. Data are means ± relative uncertainty error from three independent biological replicates. +C, growth in the presence of glucose; −C, growth without glucose.

The qualitative and quantitative changes in the most relevant lipid classes of the *A. niger* N402 ethanolic extracts were investigated using LC–ESI-MS/MS. In particular, our attention was focused on the relative changes in the lipid distributions (e.g., chain length, saturation) within three specific lipid classes: (i) free fatty acids (FFAs); (ii) lysophosphatidylcholines (lysoPCs); and (iii) diacylglycerols (DAGs). The lipid profiles and their relative molar ratios are shown in Table 1, Table 2 and Table 3, and in Figure 3, Figure 4, Figure 5, Figure 6, Figure 7 and Figure 8. 

**Table 1:** Free fatty acid profile of ethanolic extracts of samples A–D from the mycelia of *Aspergillus niger* N402, using LC–ESI-MS analysis in negative ion mode.

Extracted-Ion Chromatogram (*m/z*)	Retention Time (min)	FFA
277.3; 577.5	8.1	18:3
279.3; 581.5	10.0	18:2
255.3; 533.5	11.3	16:0
281.3; 585.5	12.7	18:1
283.3; 589.5	16.0	18:0

**Table 2:** Lysophosphatidylcholine acyl profile of the ethanolic extracts of samples A–D from the mycelia of *Aspergillus niger* N402, obtained by LC–ESI-MS analysis in the positive ion mode.

Extracted-Ion Chromatogram (*m/z*)	Retention Time (min)	Composition
518.3; 184.1	8.1	18:3
520.3; 184.1	10.3	18:2
496.3; 184.1	11.5	16:0
522.3; 184.1	13.2	18:1

**Table 3:** Diacylglycerol profile of the ethanolic extracts of samples A–D from the mycelia of *Aspergillus niger* N402, obtained by LC–ESI-MS/MS analysis in positive ion mode. The spectra signals for the [M+H]^+^, [M+Na]^+^ and [M-H_2_O+Na]^+^ fragments were taken into account for the evaluation of the % molar fractions for each extract.

Extracted-Ion Chromatogram (*m/z*)	Retention Time (min)	Composition	Acyl Chains
613.5; 635.5; 595.5	35.4	36:6	18:3/18:3
615.5; 637.5; 597.5	37.0	36:5	18:2/18:3
591.5; 613.5; 573.5	37.8	34:3	18:3/16:0
617.5; 639.5; 599.5	38.4	36:4	18:2/18:2
593.5; 615.5; 575.5	39.2	34:2	18:2/16:0
619.5; 641.5; 601.5	39.8	36:3	18:2/18:1
595.5; 617.5; 577.5	40.6	34:1	18:1/16:0
621.5; 643.5; 603.5	41.2	36:2	18:1/18:1
623.5; 645.5; 605.5	42.4	36:1	18:1/18:0

#### 2.2.1. Free Fatty Acid Determination

The profiles of the FFAs were analysed using LC–ESI-MS in negative ion mode and exploiting the high efficiency of our optimised chromatographic conditions (see Experimental Section). The corresponding chromatograms showed a series of well-separated peaks with retention times between 8.1 min and 16.0 min (Figure S1). These were associated to the mass spectra with ion signals corresponding to [M−H]^−^ and to dimeric adducts [2M−2H+Na]−, through which it was possible to infer their qualitative distributions (Table 1).

As expected, for a given chain length, the FFA retention times were shorter for the more polyunsaturated acyl chains (e.g., Table 1, C18:3 *vs.* C18:2), while for a fixed level of saturation (Table 1, C16:0 *vs.* C18:0), the FFA retention times were shorter for the shorter acyl chain. From the quantitative data, the relative molar fractions (intra-sample FFA distributions) were evaluated by assuming that all of these FFA species have the same ESI(-) response factor [20]. Overall, the data reported in Figure 3 provide the relative lipid distributions (*i.e.*, % abundances or molar fractions) within the FFAs for the given samples. Thus, *A. niger* N402 produces five major FFAs where the structural changes are restricted to the C16 and C18 chain lengths with the unsaturation ranging from 0 to 3 double bonds. The unsaturated FFAs were the dominant species, in particular the 18:2 FFA represented about 50% of all of the FFAs, while the 18:3 FFA was always the less abundant one. The total unsaturation index of these FFAs did not significantly change across samples A–D (mean, 1.3 ± 0.1), which indicates that the haemolytic activity should not be simply related to the number of unsaturated bonds within the acyl chains. In all of these samples, the 18:2 FFA was the most abundant FFA species (Figure 3, sample B in particular), while the 18:1 FFA had a significant role only in samples A and C, and the 18:0 FFA only in sample D (Figure 3). 

Although the intra-sample lipid distribution of the FFA lipid species was useful to compare their distribution within a given sample, it was necessary to have a precise quantitative parameter that can be used to relate the overall distribution of these FFAs in the four samples. Thus, we defined and evaluated the “inter-sample FFA distribution” expressed as % molar fraction of all of the lipids belonging to the FFA class, with respect to the overall amount of lipids belonging to the same class in all of the four samples (see Experimental Section for further details). When normalised with respect to the corresponding biomass obtained from the broths of samples A–D (Table S1), this quantitative parameter also indicated that the levels of total FFAs in samples A and C were significantly higher than in the corresponding samples B and D, respectively (Figure 4). Moreover, this analysis indicated that among all four of these samples, sample A contained a higher absolute amount of total FFAs than all of the other samples. Thus, even if the overall unsaturation index was almost constant across samples A–D, the increased abundances of the total FFAs in samples A and C, with respect to those of the corresponding samples B and D, demonstrate that the FFA lipid class might have an active role in determining the observed haemolytic activity. 

#### 2.2.2. Lysophosphatidylcholine Composition

The lysoPCs were analysed under the same chromatographic conditions used for the FFAs (see Experimental Section), although in this analysis, the measurements were carried out in ESI positive-ion mode. The chromatograms obtained showed a series of well-separated peaks with retention times from 8.1 min to 13.1 min (Figure S2), that were associated to the mass spectra with ion signals corresponding to [M+H]^+^ and the common phosphatidylcholine (PC) fragment ion at *m/z* 184 (Table 2). 

The intra-sample relative abundances of the lysoPCs in samples A–D from *A. niger* N402 showed that the major fatty acid chains are the same as those in the FFAs profile, although that of 18:0 was only just detectable in this lipid class (Figure 5). Similar to the FFAs, the relative molar fraction of the 18:2 FFA increased in samples B and D, while the 18:3 and 18:1 FFAs decreased, compared to samples A and C, respectively. The unsaturation indices of these lysoPC species did not significantly change across the ethanolic extracts of samples A–D (mean, 1.6 ± 0.1), again suggesting that the haemolytic activity cannot simply be related to the acyl chain unsaturation.

The inter-sample relative abundances (expressed as % molar fractions) also indicated that the lysoPCs were much lower in sample D with respect to sample C, and significantly lower in sample B with respect to sample A (Figure 6). However, sample C showed the highest amount of lysoPC class among all of the samples, in contrast with the FFAs (Figure 4) which were highest in sample A. Thus, the increased abundance of the total lysoPCs in samples A and C with respect to the corresponding samples B and D, paralleled the behaviour of the FFAs, which suggests a contribution of the lysoPCs to the observed haemolytic activity. 

#### 2.2.3. Diacylglycerol Composition

Chromatographic peaks with retention times from 35.4 min to 42.4 min were obtained for the DAGs (Figure S2), and from 49.0 min to 114.0 min for the TAGs, using the LC–ESI-MS/MS in positive ion mode, which showed the signals corresponding to the [M+H]^+^and [M+Na]^+^ ions. It was possible to define the chain lengths and the unsaturation indices for each of the acyl chains, and thus to obtain a complete profile of the DAG and TAG species. As the occurrence of the TAGs was strongly related to the growth time of the *A. niger* N402 mycelia, their changes do not represent useful markers of the haemolytic activity observed. Thus, the data for the TAGs are not reported here.

In contrast to the FFAs and lysoPCs, the DAGs not only showed a relevant redistribution of the acyl chains linked to the glycerol backbone (Table 3, Figure 7), but also their overall absolute levels significantly increased with 3 days of growth of samples B and D, with respect to 1 day of growth of samples A and C (Figure 8). Considering the intra-sample distributions (Figure 7), samples A and C showed remarkable increases in the longer and highly unsaturated acyl chains (*i.e.*, 18:2, 18:3), compared to the corresponding relative abundances in samples B and D, while the opposite trend was seen for the shorter and saturated/mono-unsaturated acyl chains (16:0, 18:1). As a final outcome of this re-distribution, not only was the mean unsaturation index of the extracts of samples A and C significantly greater than that for the extracts of samples B and D (2.9 ± 0.1 *vs.* 2.4 ± 0.1, respectively), but also the mean chain lengths of the FFAs present in the extracts of samples A and C were longer than in those of samples B and D. 

The quantitative analysis of the inter-sample distribution (Figure 8) indicated that the amounts of the total DAGs significantly increased in the extracts of samples B and D, with respect to those of samples A and C. Although the DAGs are not known in the current literature as haemolytically active lipid species, the essentially opposite changes shown by the DAGs in comparison to FFAs and lysoPCs appear to indicate an active, although indirect, role of the DAGs towards the haemolytic activity, such as, e.g., their serving as a reservoir for the FFAs.

#### 2.2.4. Glycerophospholipid Composition

The PC, phosphatidylethanolamine and phosphatidylinositol profiles were also obtained by analysis with the same LC–ESI-MS/MS positive ion mode conditions used for the lysoPCs (see Experimental Section). However, the relative changes observed in the extracts of samples A–D for the total lipids within these three GPL classes were not significantly different. For this reason, and because the GPLs are not haemolytic, they were not investigated further here. 

### 2.3. Haemolytic Activity of Commercial Free Fatty Acids and Lysophosphatidylcholine Species Associated with Ethanolic Extracts of Aspergillus niger N402

**Figure 9:**
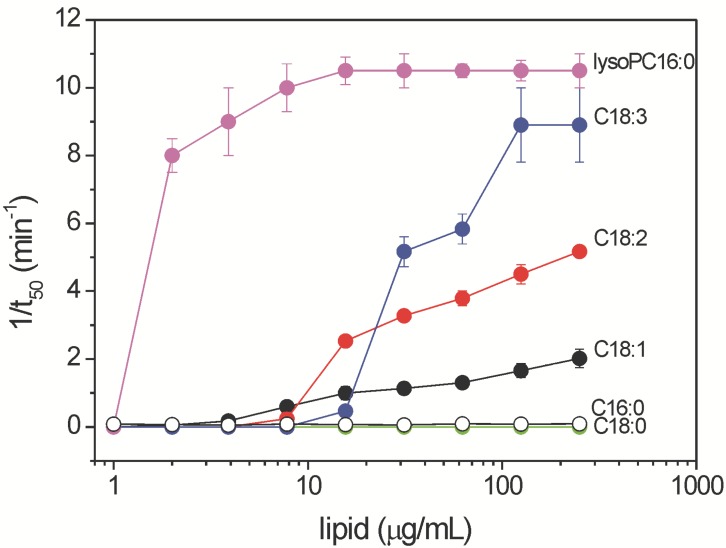
Dependence of the half-time of haemolysis (1/t_50_) on the concentrations of free fatty acids and lysophosphatidylcholine 16:0. t_50_, time required to induce 50% lysis of bovine erythrocytes. Data are means ± standard error from three independent biological replicates.

To further explore the haemolytic potential of FFAs and lysoPCs detected in ethanolic extracts of *Aspergillus niger* N402, several commercial FFAs and 16:0 lysoPC were assayed with bovine erythrocytes (Figure 9). The haemolytic activities here were mainly associated with the unsaturated FFAs. For a given chain length, these haemolytic activities increased with the degree of unsaturation (C18:1 < C18:2 < C18:3). Saturated FFAs did not have any significant haemolytic activities, and only minor haemolysis can be observed at relatively high (>100 μg/mL) concentrations of the short-chain FFAs, such as C12:0 and C14:0. LysoPC 16:0 is even more haemolytically active than the 18:3 FFA, which indicates that in addition to the unsaturation state of the acyl chain, the size of the lipid headgroup might also be important for haemolytic activity.

### 2.4. Discussion

From the lipid profiles obtained in the present study, and from the haemolytic activity tests of commercial FFAs and lysoPC, it appears that the haemolytic activity observed for the ethanolic extracts of samples A–D from *A. niger* N402 is related to the changes in (i) the intra-sample distributions within each class; and (ii) the overall amounts (inter-samples relative changes) of total FFAs and total lysoPC lipid species. 

FFAs are known to be involved in several functions in fungi, and they have been shown to inhibit the growth of microbial competitors [21]. The oxygenated poly-unsaturated fatty-acid derivatives known as the oxylipins have been shown to have functions in fungal colonisation of plant hosts [22] and in the development and sporulation of filamentous fungi [23]. Also, the fatty-acid profiles can be used as a taxonomic tool to discriminate between different *Aspergillus* spp. [24,25]. At the same time, the high concentrations of FFAs in fungi have been reported to induce haemolytic and cytolytic effects. Investigations of ethanolic extracts of the xerotolerant mycotoxigenic fungus *Wallemia sebi* revealed that the unsaturated FFAs are responsible for the haemolytic activity of these extracts, and suggested the potential involvement of the FFAs in the formation of lesions in subcutaneous infections, in farmer’s lung disease, and in the consumption of food and feed that are contaminated with food-borne *W. sebi* [18]. Furthermore, the haemolytic activity of the fungus *Chaetomium spiculipilium* has been reported to be associated with the production of extracellular FFAs [19]. Examples of toxic FFAs can also be found in other organisms. Poly-unsaturated FFAs extracted from the microalgal species *Fibrocapsa japonica* [26] and the dinoflagellate *Cochlodinium polykrikoides* [27] have been reported to be haemolytic and responsible for icthyotoxicity through their accumulation in fish, and for the haemolysis and cytotoxicity induced by eggs of the common barbel(*Barbus barbus*) [20]. 

The FFA profiles of the ethanolic extracts of samples A–D from *A. niger* N402 mycelia obtained in this study are in line with previous analyses of different *Aspergillus* spp., which have showed that C18:2, C18:1 and C18:3 are the most abundant FFA species [28]. These FFAs have also been detected as the most abundant (usually comprising up to 80% of the fatty acids) in the mycelia [29,30,31,32,33,34] and spores [35,36] of several commercially important *A. niger* strains. For the unsaturation indices of the lipid classes investigated in the present study, the FFAs were characterised by an unsaturation index of 1.3 ± 0.1, which is essentially independent of the growth conditions of the *A. niger* N402 mycelia. 

The haemolytic activity of these ethanolic extracts of samples A–D from *A. niger* N402 might also be partially related to the intra-sample and inter-sample changes in the lysoPC profile. Indeed, several saturated and unsaturated lysoPCs containing C12-C18 acyl chains have been shown to cause strong haemolysis of rabbit erythrocytes under *in vitro* conditions [37]. The data from the present study specifically indicate that the extracts with the highest haemolytic activities (samples A and C) indeed contained the highest levels of lysoPCs, although as the absolute amounts of FFAs were much higher (almost twice) in sample A than sample C, the haemolytic activity (Figure 2) appears to be mainly related to the FFA content. By using external calibration based on known amounts of commercially available quantitative standard PC 17:0/20:4, and assuming the same ionisation efficiency for lysoPC, we estimated that the overall lysoPCs in samples A and C were 7 ± 1 μg/mL and 11 ± 1 μg/mL, respectively. According to Figure 9, both of these values suggest that lysoPC in *A. niger* can have relevant haemolytical activity. Similarly to the FFAs, the unsaturation index of these lysoPCs was almost unchanged (1.6 ± 0.1), regardless of the growth conditions. 

Finally, we should emphasise that changes in the lipid profiles among the extracts of samples A–D were also seen for the DAG species. From these data, the greatest changes would appear to be mainly accounted for by the time-rewiring of the 36:2 (18:1/18:1) and 36:1 (18:1/16:0) DAGs in the extracts of samples B and D, to the 36:4 (18:2/18:2) DAG in the extracts of samples A and C. The increased % molar fraction of total DAGs in extracts obtained after growth of the fungi under carbon-depriving conditions might point to enhanced catabolism of lipids and use of membrane lipids as an alternative source of carbon under these conditions. Recent transcriptome and secretome studies of *A. niger* [38] and *A. nidulans* [39] have indeed shown that upon carbon starvation, there is clear down-regulation of synthases, and pronounced up-regulation of hydrolases involved in lipid metabolism. 

The effects of different cultivation conditions on the *A. niger* lipid composition during growth have already been investigated [29,30]. These previous studies showed that prolonged growth in manganese-deficient medium significantly lowers the lipid levels in the mycelia [29] and the protoplasts [30] of the citric acid-producing *A. niger* B60 strain, although this did not alter the ratios of the major saturated and unsaturated fatty acids. In the present study, *A. niger* N402 grown under different conditions appears to retain almost unchanged overall unsaturation indices for their FFAs and lysoPCs, but not those for their DAGs. Lower temperatures have also been shown to increase FFA unsaturation in mycelia of the *A. niger* VTT-D-77020 strain, as analysed during their exponential phase of growth [40]. Moreover, the effects of temperature and different times of cultivation on lipid compositions were explored in the citric acid-producing *A. niger* VKM F-34 strain [41]. The fatty acid composition analysed showed no significant temperature-dependence, but the FFA unsaturation was significantly higher after 24 h compared with 48 h of cultivation. In contrast, there were no significant differences in the unsaturation index in mycelia of the *A. niger* A60 [31] and A-138 [32] strains after prolonged times of cultivation (3–8 days). Furthermore, Singh [42] did not find any significant differences in the FFA compositions in the cellulolytic *A. niger* AS-101 strain grown for 6 days on different carbon sources. In our case, a similar composition was detected, especially for the unsaturated FFAs, regardless of the time of growth and the presence or absence of glucose in the medium. However, the FFA profile did change according to these two variables. 

## 3. Experimental

### 3.1. Materials

Lauric (C12:0), myristic (C14:0), palmitic (C16:0), palmitoleic (C16:1), stearic (C18:0), oleic (C18:1), linoleic (18:2) and α-linolenic (C18:3) acids, and absolute ethanol, were from Sigma Aldrich (St. Louis, MO, USA). 1-Palmitoyl-2-hydroxy-*sn*-glycero-3-phosphocholine (C16:0 lysoPC) was from Avanti Polar Lipids (Alabaster, AL, USA). 

### 3.2. Fungal Strain and Growth Conditions

The *A. niger* N402 strain used in this study is a mutant with short conidiophores (*cspA1*) that was derived from the wild-type strain (CBS 120.49, ATCC 9029) [43]. Submerged cultures were grown in minimal or complete media (minimal medium supplemented with 0.5% [w/v] yeast extract, 0.2% [w/v] casamino acids and vitamins) [44]. For spore preparation, *A. niger* N402 was grown on malt extract agar (Blakeslee’s formula, using phyton instead of peptone) at 30 °C for 4 days. The spores were collected in sterile 0.9% (w/v) NaCl and inoculated into 100 mL complete medium, with 10^7^ spores mL^−^^1^. The cultures were grown overnight, and then the whole biomass was transferred into 50 mL minimal medium either with 1% (w/v) glucose as carbon source or without a carbon source. The submerged cultures were grown in 500 mL Erlenmeyer flasks at 30 °C on a rotary shaker at 180 rpm, for 24 h or 72 h.

### 3.3. Preparation of Ethanolic Extracts

The whole fungal biomasses (average wet biomasses: sample A, 5.7 g; sample B, 3.9 g; sample C, 3.7 g; and sample D, 1.7 g, Table S1) were harvested by filtration, washed with sterile 0.9% (w/v) NaCl, dried, and frozen in liquid nitrogen. All of the tissue was ground to a fine powder with a mortar and pestle. Absolute ethanol (20 mL) was added to each of these samples, and the extractions were left overnight at room temperature. The resulting ethanolic extracts were filtered to remove the remnants of the fungal tissue. The solvent was completely evaporated from the resulting filtrates. The dried deposits from these extracts were then resuspended in 1 mL absolute ethanol, and centrifuged at 34,882 ×*g* at 25 °C for 10 min. Finally, the supernatants were transferred into glass vials to a final volume of 1 mL (with absolute ethanol).

### 3.4. Haemolytic Assay

The haemolytic activity was measured on a kinetic microplate reader (Synergy Mx, BioTek Instruments, Winooski, VT, USA), as described previously [45]. The mycelial extracts and the commercial fatty acids or lysophosphatidylcholine were diluted progressively in absolute ethanol, and 25 μL of the resulting solutions (or pure ethanol) were added to 175 μL bovine erythrocyte suspensions in erythrocyte buffer (140 mM NaCl, 20 mM Tris-HCl, pH 7.4) with an apparent absorbance of 0.5 at 630 nm. The decrease in the apparent absorbance was measured for 30 min, at 20 s intervals, to determine the t_50_; *i.e.*, the time necessary for 50% haemolysis of the bovine erythrocytes. The haemolytic activity is expressed as 1/t_50_ (min^−^^1^). All of the experiments were performed at 25 °C, with each measurement repeated at least three times. The solvent was not haemolytic in itself. 

### 3.5. LC–ESI-MS/MS Analysis

The LC–ESI-MS/MS was performed using a Hewlett-Packard Model 1100 series liquid chromatograph coupled to both an Agilent 1100 photo-diode-array detector and a Bruker Esquire-LC quadrupole ion-trap mass spectrometer, equipped with an atmospheric pressure electrospray ion source. Each family of lipids was separated under the following LC conditions: Zorbax Eclipse XDB-C8 3.5 µm column; mobile phases: pump A, MeOH/H_2_O (7:3; v/v) with 28 mM ammonium acetate; pump B, MeOH with 12 mM ammonium acetate; gradient, 35% to 100% B over 40 min; flow rate, 0.8 mL/min; injection volume, 10 μL ethanolic solution (of 1.0 mL of each extract). The measurements were carried out in two replicates. High-purity nitrogen was used as the nebuliser, at 35 psi, and also as the drying gas, at 300 °C, at a constant flow rate of 7 L/min. Full scan spectra (50–1000 Da) were acquired in negative ion mode for FFAs, phosphatidylethanolamine and phosphatidylinositol, and in positive ion mode for lysoPCs, PC, DAGs and TAGs. The structural information for the compositions was obtained from the in-source collisional induced dissociation fragments on the selected precursor ions. Data Analysis, version 3.0 (Bruker Daltonik GmbH, Bremen, Germany) was used to analyse the mass spectra. The extracted ion current was integrated from the total ion current for the quantitative analysis of each component in a specific lipid class, on the assumption that lipids belonging to the same lipid class should have a similar efficiency of ionisation. 

The intra-sample lipid distributions were expressed as % relative abundance of each lipid species within a given lipid class (L_nm_, where *n* represent the lipid species and *m* the lipid class) and were evaluated from the ratio of the area of the corresponding extracted ion current (A_n_) with respect to the overall area of all of the species belonging to a given class and a given sample; *i.e.*, through the equation:

L_nm_ (%) = [A_n_/∑_n_ A_n_]_m_ × 100 (1)

Thus, for example, the 42% relative abundance of FFA 18:2 in sample A as reported in Figure 4 was evaluated by the ratio of the area of the extracted ion current [M−H]^−^ at *m/z* 279 to the summation of the areas of all of the detected and extracted ion currents at *m/z* 277 (18:3), 279 (18:2), 281 (18:3), 283 (18:0) and 255 (16:0). The % relative uncertainty error was within the range of 3% to 5% for all of the detected lipid species. 

The unsaturation index of a given lipid class (UI_m_) was calculated as:

UI_m_ = [∑_n_ L_nm_ (%) × unsaturation number]/100 (2)

The inter-sample distributions were expressed as % molar fractions of all lipids belonging to a given class (total FFAs, or lysoPCs, or DAGs), with respect to the overall amount of lipids belonging to the same class in all of the four samples. This was evaluated by the ratio of the total area (A_tot_ = Σ_n_ A_n_), after normalisation to the lyophilised biomass of each sample, to the summation of the areas of all of the species belonging to a given class *m* in a given sample *r*; *i.e.*, through the equation:

Total L_nm_ (%) = [(A_tot_)_r_/∑_r _ (A_tot_)_r_]_m_ × 100 (3)

Thus, for example, the 55% molar ratio of total FFAs in sample A as reported in Figure 4 was evaluated by the ratio of the total corrected area for FFAs detected in sample A, to the total corrected areas for FFAs detected in all of the four samples A–D. The % relative uncertainty error for these measurements was evaluated to be about 7% for all of the investigated lipid classes (FFAs, lysoPCs and DAGs). 

## 4. Conclusions

Ethanolic extracts of samples A–D from *A. niger* N402 mycelia have haemolytic activity towards bovine erythrocytes. Essentially, this haemolytic activity diminished with the time of growth of the *A. niger* N402 mycelia, and also when the *A. niger* N402 mycelia were grown in carbon-depleted medium. The LC–ESI-MS/MS analyses for the lipid compositions of these extracts and the haemolytic assays of commercial lipids strongly suggest that this bioactivity is related to the abundance of the unsaturated acyl chains (mainly 18:1 and 18:3) and is metabolically regulated through three different lipid classes, the FFAs, lysoPCs and DAGs. Moreover, the overall abundance of FFAs and lysoPCs was higher in the extracts showing the highest haemolytic potential. 

## Supplementary Materials

Supplementary materials can be accessed at: http://www.mdpi.com/1420-3049/19/7/9051/s1.
