# Supplementary material for: Targeted Lipid Analysis of Haemolytic Mycelial Extracts of Aspergillus niger
Source: Molecules. 2014 Jun 30;19(7):9051–69. doi: 10.3390/molecules19079051 (PMC6272009; doi:10.3390/molecules19079051)

Supplementary Materials

Table S1. Wet biomasses of *Aspergillus niger* N402 strain grown at 30 °C for 24 or 72 h in minimal medium supplemented with 1% (w/v) glucose as a carbon source or without a carbon source on a rotary shaker at 180 rpm; the amount of total solids extracted from crushed mycelium after an overnight extraction in absolute ethanol; and average pH of the growth medium after 24 or 72 h. Data are presented as mean ± SD from three independent biological replicates. **MM**—minimal medium, **+C**—with glucose as carbon source, **−C**—without carbon source.

	Average Wet Biomass (g)	Average Total Solids (mg)	% of Isolated Solids	Average pH of Medium
**N402 MM+C 24h**	5.7 ± 0.3	44 ± 10	0.77	3.6 ± 0.1
**N402 MM+C 72h**	3.9 ± 0.5	18 ± 3	0.46	5.5 ± 0.2
**N402 MM−C 24h**	3.7 ± 0.7	33 ± 3	0.91	4.4 ± 0.4
**N402 MM−C 72h**	1.7 ± 0.4	9 ± 3	0.53	5.7 ± 0.1

Figure S1. TIC chromatogram as obtained by full-scan LC-ESI-MS in negative ion mode for extract A as an example, from where data for FFAs (in the range 8.1÷16 .0 min, reported in Table 1 and Figure 3) have been derived.

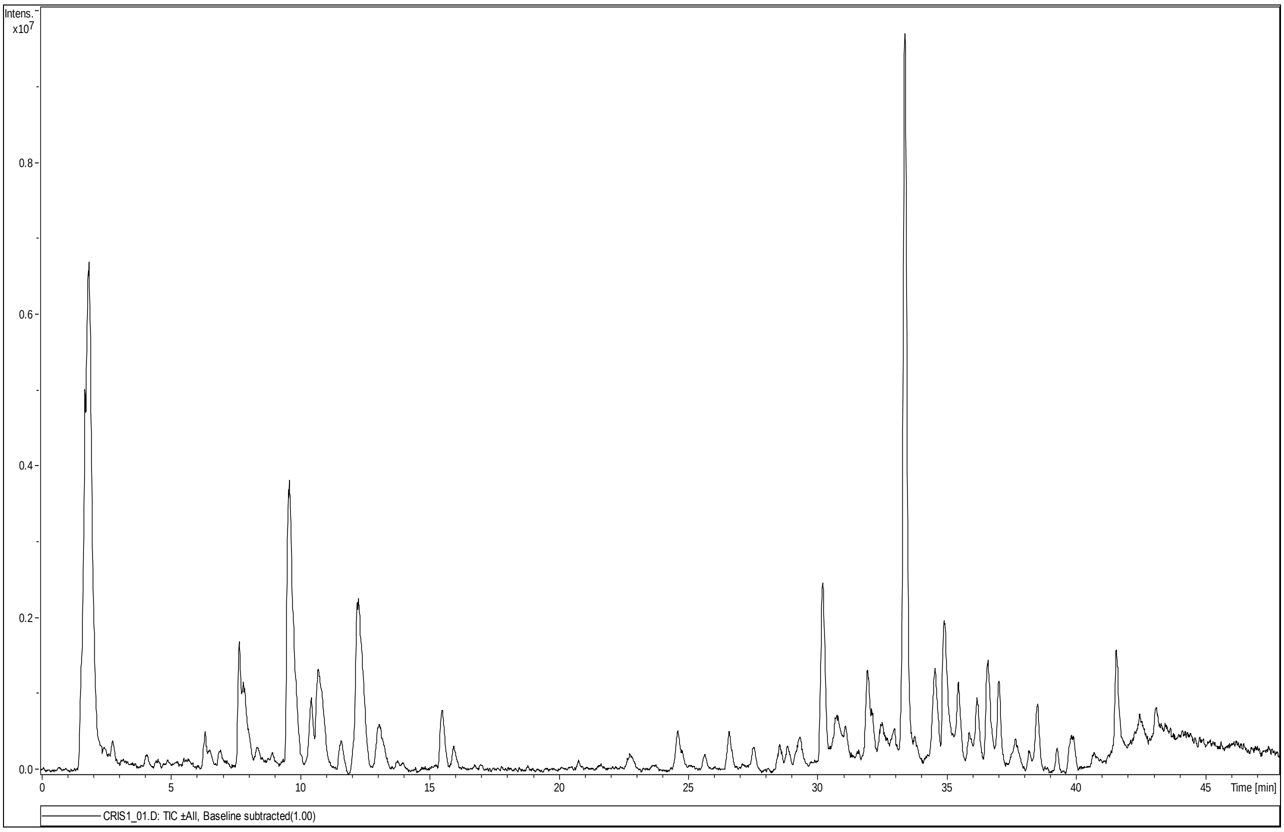

Figure S2. TIC chromatogram as obtained by full-scan LC-ESI-MS in positive ion mode for extract A as an example, from where data for LysoPC (in the range 8.1÷13.2 min, reported in Table 2 and Figure 5) and for DAGs (in the range 35.4 ÷42.4 min, reported in Table 3 and Figure 7) have been derived.

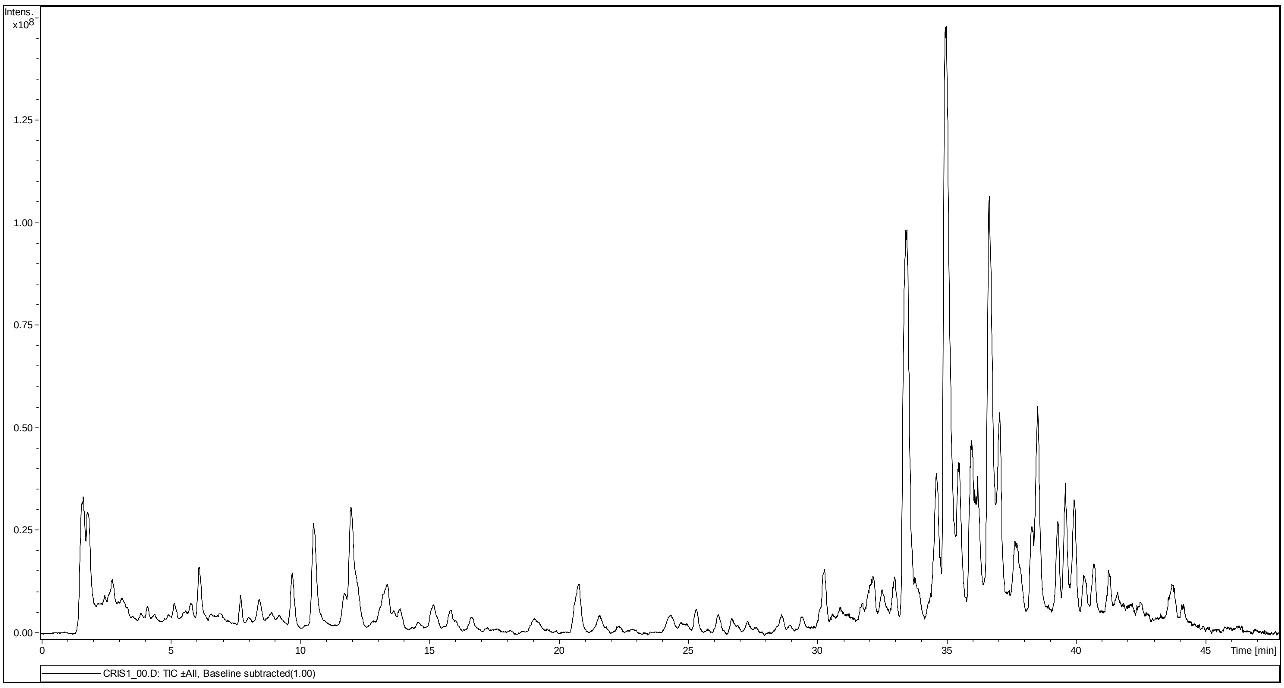

Supplement: Supplementary file 1 [file molecules-19-09051-s001.pdf]
